# A Novel Long-range Excitatory Neural Circuit from the Magnocellular Red Nucleus to Spinal Dorsal Horn Facilitates Neuropathic Pain-like Behaviors in Male Mice

**DOI:** 10.1007/s12264-025-01553-7

**Published:** 2025-12-24

**Authors:** Jiali Shi, Yinfeng Yuan, Yuhao Luo, Yue Guo, Jiashu Lian, Lin Lin, Danni Chen, Qian Wang, Xiumin Xue, Zhichao Chen, Yongjie Wang, Zhihui Huang

**Affiliations:** 1 School of Pharmacy, Hangzhou Normal University, Hangzhou, 311121 China; 2 Department of Pharmacy, Sir Run Run Shaw Hospital, Zhejiang University School of Medicine, Hangzhou, 310016 China; 3 Department of Ultrasound, Sir Run Run Shaw Hospital, Zhejiang University School of Medicine, Hangzhou, 310016 China

**Keywords:** Neuropathic pain, Magnocellular red nucleus, Spinal dorsal horn, Neural circuit, Optogenetics, Chemogenetics

## Abstract

Neuropathic pain arises from a primary lesion or disease affecting the somatosensory system; however, the brain circuitry underlying the regulation of neuropathic pain remains unclear. The red nucleus (RN) is a critical brain region involved in regulating muscle tension, coordinating movement, and facilitating sensorimotor integration. While some evidence has shown that RN regulates pain, its specific role in neuropathic pain and associated neural circuits remains elusive. In this study, we found that CaMKIIα-positive neurons in the magnocellular red nucleus (RMC^CaMKIIα^) were activated following common peroneal nerve (CPN) ligation-induced chronic neuropathic pain in adult male mice. Interestingly, chemogenetic and optogenetic inhibition of these RMC^CaMKIIα^ neurons alleviated the thermal hyperalgesia and mechanical allodynia in the neuropathic pain model mice, whereas activation of these neurons sufficiently induced the mechanical allodynia and thermal hyperalgesia in naïve mice. Trans-synaptic viral tracing studies further revealed that long-range CaMKIIα^+^ neuron projections from RMC to the dorsal horn (DH) facilitated the neuropathic pain-like behaviors following CPN ligation. DH neurons received direct innervation from RMC^CaMKIIα^ neurons, and inhibition of RMC^CaMKIIα^-DH^CaMKIIα^ circuits alleviated the neuropathic pain in the ligated mice. Taken together, these results identify a novel long-range excitatory neural circuit from RMC to DH that facilitates the neuropathic pain-like behaviors in adult male mice, providing a new target for neuropathic pain treatment.

**Supplementary Information:**

The online version contains supplementary material available at 10.1007/s12264-025-01553-7.

## Introduction

Neuropathic pain is triggered by primary lesions or disease of the somatosensory nervous system [1, 2], and is characterized by abnormal hypersensitivity to noxious stimuli (hyperalgesia) and nociceptive responses to non-noxious stimuli (allodynia) [3]. Both noxious and innocuous stimuli are pathologically amplified under neuropathic pain conditions. Its prevalence in the general population is estimated at 7%−8%, accounting for 20%−25% of individuals with chronic pain [4]. Neuropathic pain is challenging to treat and severely impacts patients’ quality of life. Thus, it is pivotal to unravel neurobiological mechanisms to provide novel targets for neuropathic pain.

Neurophysiological studies have identified several brain regions activated during pain processing, including the anterior cingulate cortex (ACC), prefrontal cortex (PFC), periaqueductal grey (PAG), insula, somatosensory cortex, and cerebellum [5–7]. Brain networks, involving pathways descending from the cerebral cortex to the spinal cord, can modulate afferent pain signals through inhibition or excitation at multiple levels [8, 9]. Red nucleus (RN), located in the ventral midbrain, is cytoarchitecturally divided into the magnocellular (RMC) and parvicellular (RPC) parts [10–13]. The axons of efferent neurons in RMC, after crossing the midline, project mainly to the spinal cord, whereas RPC neurons send their major projections to the inferior olive [10, 14]. RN is primarily known for regulating muscle tension, coordinating movement, and facilitating sensorimotor integration [15, 16]. However, emerging evidence has suggested that it is also involved in pain regulation. Recent studies have identified that RN dual-directionally modulates the onset and persistence of neuropathic pain through secreting pro- and anti-inflammatory cytokines [17, 18]. Functional imaging studies indicate that rubral activation mainly correlates with motor performances with a complex rhythm, sensory discrimination, overt speech production, pain processing, and procedural complexity, whereas limited correlation is observed during simple movement performance [19]. Although these studies have provided evidence of the RN’s functions in pain regulation, its detailed roles in neuropathic pain and the associated neural circuits remain elusive.

In the present study, we found that CaMKIIα-positive neurons in the magnocellular red nucleus (RMC^CaMKIIα^) were activated in male mice with neuropathic pain. Chemogenetic and optogenetic inhibition of RMC^CaMKIIα^ neurons alleviated the neuropathic pain in model mice, while activation of these neurons induced the neuropathic-like behaviors in naïve mice. Mechanistically, trans-synaptic viral tracing and functional studies identified a novel long-range excitatory neural circuit from RMC to dorsal horn (DH) that was responsible for neuropathic-like behaviors in mice after common peroneal nerve (CPN) ligation. These results provide evidence, for the first time, that a novel neural circuit from RMC to the DH pathway facilitates neuropathic pain in male mice.

## Materials and Methods

### Animals

Fos-Cre^ERT2^; Ai14 mice were obtained by crossing transgenic Fos-Cre^ERT2^ mice (Shanghai Model Organisms Center, Shanghai, China) with Ai14 mice (Cre-dependent tdTomato reporter, Shanghai Model Organisms Center, Shanghai, China). tdTomato expression was induced in Fos-Cre^ERT2^; Ai14 mice by 4-hydroxytamoxifen (4-OHT) treatment [50 mg/kg, MedChemExpress (MCE), New Jersey, USA] intraperitoneally (i.p.). Both Fos-Cre^ERT2^ and Ai14 mice were maintained under the background of the C57BL/6 strain, and genotyping of these mice was performed by polymerase chain reaction (PCR). Adult male mice were used for all experiments in this study. The mice were housed under standard laboratory conditions (temperature 22 ± 2°C and humidity 50%−60%) in the Laboratory Animal Centre of Hangzhou Normal University. The use of animals and all relevant experimental protocols were approved by the Animal Care and Use Committee of Hangzhou Normal University (HSD20210106).

### Stereotaxic Injection

All animals were under 4% isoflurane anesthesia (R510-22-10, RWD, Shenzhen, China), and then were fixed on a stereotaxic device (RWD, 68045). Viruses were injected into RMC [anteroposterior (AP): −3.4 mm; mediolateral (ML): ±0.75 mm; dorsoventral (DV): −4.35 mm] with a volume of 100−150 nL/site at a rate of 20 nL/min using an air pressure system connected to a glass pipette (BF100-78-10, Sutter Instrument, CA, USA). Ten minutes after viral injection, the glass pipette was withdrawn slowly to avoid backflow of viruses. The primary viruses included AAV-hSyn1-GCaMP6s-P2A-nls-dTomato (AV9004, Vigene, Jinan, China), AAV9-CaMKIIα-hM3D(Gq)-mCherry (AV202003-AV9, Vigene), AAV9-CaMKIIα-hM4D(Gi)-mCherry (AV202012-AV9, Vigene), AAV9-CaMKIIα-eNpHR3.0-EYFP (AV201019-AV9, Vigene), AAV9-CaMKIIα-hChR2-mCherry (AV201002-AV9, Vigene), AAV9-EF1α-DIO-hM4D(Gi)-mCherry (AV202014-AV9, Vigene), AAV9-CaMKIIα-mCherry (AV200069-AV9, Vigene), AAV-hSyn-taCasp3-T2A-TEVp-P2A-EGFP-3xFLAG-WPRE (H13919, OBiO, Shanghai, China), AAV-hSyn-MCS-EGFP-3xFLAG (AOV062, OBiO), AAV2-retro-hSyn-Cre-P2A-GFP (AV204032-AV2 retro, Vigene), and scAAV2/1-hSyn-Cre-pA (S0292-1, Taitool, Shanghai, China). For chemogenetic manipulation, clozapine-N-oxide (CNO) (1.5 mg/kg, MCE) was dissolved in normal saline and was injected i.p. or intrathecally (i.t.) 30 min before behavioral testing once per day for 6 days.

### Optical Fiber Implantation

Optical fiber implantation was performed as described previously [20]. Mice were anesthetized with 4% isoflurane and mounted in a stereotaxic apparatus. The skull was exposed by midline scalp incision, and a craniotomy was performed unilaterally for introduction of an optical fiber (Newdoon, Hangzhou, China, ferrule O.D.: 1.25 mm, fiber core: 200 μm, length: 5.0 mm) into RMC (AP:  −3.4 mm; ML:  ± 0.75 mm; DV:  − 4.35 mm). Fibers were attached to the skull with dental cement and connected to a laser source using an optical fiber sleeve. For optogenetic manipulation in awake behaving mice, blue and yellow laser power was calibrated to 10 mW and 20 mW at 20 Hz for 10 min with 10 ms pulse duration, respectively (Inper, Hangzhou, China). Mice with missed injections or cannula dislocations were excluded. Control mice underwent the same procedures and received the same intensity of laser stimulation. Ca^2+^ transient signal was detected by a fiber photometry system (Newdoon).

### CPN Ligation Surgery

CPN surgery to induce chronic neuropathic pain was performed as described previously [21]. Briefly, the left common peroneal nerve between the anterior and posterior groups of muscles was ligated slowly with a 5-0 chromic gut suture until twitching of the digits was observed.

### Measurement of Mechanical Allodynia and Thermal Hyperalgesia

Mechanical allodynia was assessed by measuring paw-withdrawal threshold (PWT) using Von Frey filaments (0.008−1.0 g) to gently stroke the lateral aspect of the injured hindpaw. The percentage of withdrawal for the ipsilateral paw (nerve-injured side) was considered a hypersensitivity response. Chaplan [22] and colleagues adapted the statistical insights of Dixon to an equation for the calculation of 50% withdrawal thresholds :

$$ {5}0\% \,{\mathrm{g}}\,{\text{threshold = }}\left[ {{1}0^{(X_{f} + k\delta )} } \right]/{1}0,000 $$

The *k* value was provided by the Dixon statistics table. *δ* was the mean difference in log units between stimuli. *X*_*f*_ was the value (in log units) of the last Von Frey filament applied. If the lowest or highest filament was reached with no breaking point, the value of the lowest or highest filament was assigned.

Thermal hyperalgesia was determined by measuring latency in seconds of a withdrawal response to radiant heat applied to the center of the plantar surface of the hindpaw. Mice were placed on an elevated glass platform and allowed to habituate for 1 h. The thermal stimulus with a radiant heat source (infrared intensity of 30) was applied three times on each paw, and consecutive applications of radiant heat were separated by at least 5 min. A cutoff time of 20 s was imposed to prevent tissue damage. The latency of response for each hindpaw was defined as the average of three trials.

### Open Field Test

Open field test was performed as described previously [21]. The open field arena was a square box with opaque plexiglas walls. Each mouse was placed in an open field chamber and allowed to explore for 5 min freely. The apparatus was cleaned using 75% ethanol after each exploration. Each mouse was placed in the center of the box and recorded by a camera attached to a computer. The movement of mice was automatically tracked by a digital video camera. Time and entries in the central zone during the test were measured by EthoVision XT (Noldus, Wageningen, Netherlands) or ANY-maze software (Stoelting, Wisconsin, USA). The apparatus was cleaned using 75% ethanol after each exploration.

### Elevated Plus Maze Test

Elevated plus maze test was performed as described previously [21]. The maze was made of two open arms and two identical closed arms, which crossed each other vertically, and the instrument was equipped with a special surveillance camera for animal behavior experiments. Each mouse was placed in the center area with its head toward one open arm, and allowed to explore the maze for 5 min, recorded with a digital video camera. Time in open arms, and entries into open and closed arms were analyzed with ANY-maze or EthoVision XT software. The apparatus was cleaned using 75% ethanol after each exploration.

### Immunostaining

Mice were perfused with 1× PBS, followed by 4% paraformaldehyde (PFA). After fixation, the brain and spinal cord tissues were removed and placed in 4% PFA overnight for further fixation. For gradient dehydration, tissues were immersed in 15% and 30% sucrose solutions on the following day. The tissues were then frozen with optimal cutting temperature (OCT, SAKURA, USA) compound and sectioned into 20 µm slices. Frozen sections were blocked in 1× PBS with 5% normal goat serum (NGS) and permeabilized with 0.3% Triton X-100 for 1 h at room temperature. The sections were then incubated with primary antibodies, including c-Fos antibody (1:500, ab222699, Abcam, Cambridgeshire, UK) or CaMKIIα antibody (1:100, MA1-048, Thermo Fisher, Massachusetts, USA) overnight at 4°C. The next day, sections were washed three times and incubated with the appropriate secondary antibody at room temperature for 1 h. Secondary antibodies, including donkey anti-rabbit 546 (1:1,000, A10040, Thermo Fisher), donkey anti-mouse 488 (1:1,000, A21202, Thermo Fisher), and DAPI (1:1,000, C0060, Solarbio, Beijing, China, for nuclear staining), were applied for 1 h at room temperature. Images were obtained with microscopes (VS200 or FV3000, Olympus, Tokyo, Japan).

### Electrophysiological Recordings

All recordings were performed at room temperature. Coronal acute RMC slices (260 μm) were prepared from anesthetic mice using a vibrating tissue slicer (VT1200S; Leica Biosystems, Illinois, USA) in ice-cold standard artificial CSF (aCSF) containing (in mmol/L: 122 NaCl, 2 KCl, 2 CaCl_2_, 1.25 NaH_2_PO_4_, 26 NaHCO_3_, 10 D-glucose, and 2 MgSO_4_, pH 7.2−7.4, osmolality 300−310 mOsm) bubbled with 95% O_2_/5% CO_2_. Slices were transferred immediately to an incubation chamber containing a 32°C aCSF solution. After recovery for at least 1 h, slices were placed in a submerged recording chamber that was perfused with aCSF. Chemical reagents were from Sigma (St Louis, USA). All recordings were performed at a voltage-clamp pattern, and RMC neurons were held at  − 70 mV. Patch-clamp electrodes were pulled from borosilicate glass capillaries *via* Sutter Instrument P-97, and had resistances in the range of 5−7 MΩ. Miniature excitatory postsynaptic currents (mEPSCs) were recorded in the presence of 10 μmol/L bicuculline to block GABA_A_ receptor-mediated inhibitory currents and 1 μmol/L tetrodotoxin to block action potentials. The pipette solution for mEPSCs recording contained (in mmol/L) 105 K-Gluconate, 30 KCl, 2 MgCl_2_·6H_2_O, 5 QX-314, 10 HEPES, 0.3 EGTA, 4 Mg-ATP, 0.3 Na_2_GTP, and 10 phosphocreatine. The amplitude and frequency of mEPSCs of each neuron within 3 min were calculated. The liquid junction potential was adjusted during recording. Action potentials were recorded in current-clamp mode (*I *= 0 pA) by injecting a series of step currents (starting at  − 40 pA, lasting for 100 ms, with a delta level of 20 pA). Recordings were excluded from analysis if series resistance, input resistance, or holding current varied by  ~ 15% over the course of an experiment. Signals were filtered at 2 kHz with a MultiClamp 700B amplifier (Molecular Devices, Palo Alto, USA). Data were sampled at 10 kHz and analyzed using ClampFit 10 (Molecular Devices) or MiniAnalysis (Synaptosoft, Decatur, USA).

### Statistical Analysis

Statistical analysis was performed by one-way or two-way analysis of variance (ANOVA) followed by Bonferroni’s test for multiple comparisons, and an unpaired *t*-test for two-group comparisons. We also examined the interaction between treatments and groups. Statistical significance was accepted at the level of *P* <0.05, with asterisks in figures denoting *P-*values as follows: ^*^P <0.05, ^**^P <0.01. All data were analyzed and plotted using GraphPad Prism 8.2.1 software (GraphPad Software, San Diego, USA).

## Results

### RMC^CaMKIIα^ Neurons Were Activated in Male Mice with Neuropathic Pain After CPN

**Fig. 1:**
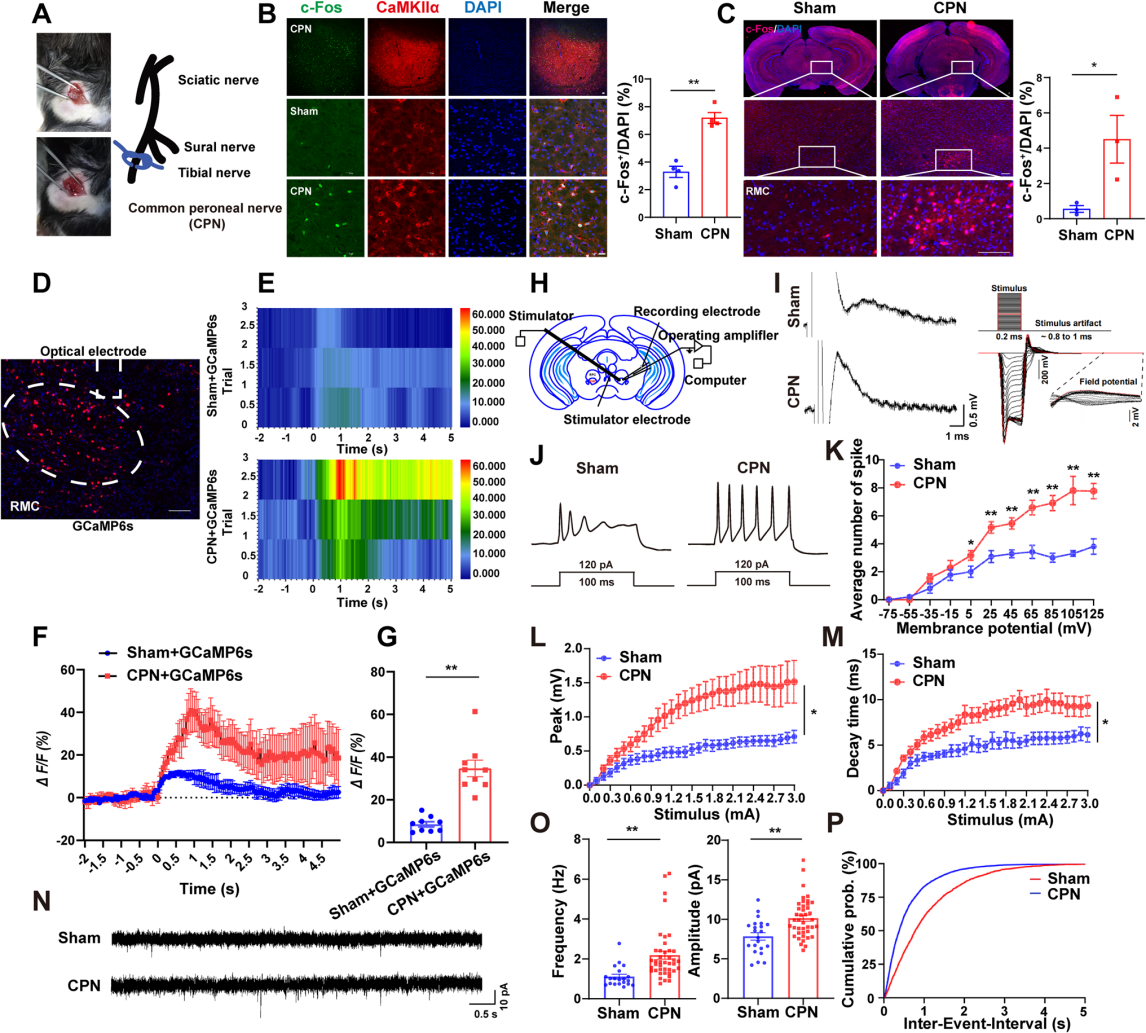
RMC^CaMKIIα^ neurons were activated in male mice with neuropathic pain after common peroneal nerve (CPN) ligation. **A** Schematic of the CPN model. **B** Image showing c-Fos (green) co-labeled with CaMKIIα (red) in RMC of male mice after CPN ligation and quantification of the percentage of c-Fos^+^ neurons (*n *= 4 mice). Scale bar, 20 μm. **C** Representative images of c-Fos^+^ (red) neurons in the magnocellular red nucleus (RMC) after sham and CPN in Fos-Cre^ERT2^;Ai14 transgenic male mice and quantification of the percentage of c-Fos^+^ neurons (*n *= 3 mice). Scale bar, 100 μm. **D** Representative photomicrographs of the viral injection site in RMC. Scale bar, 100 μm. **E** Heatmap of calcium signals recorded in RMC neurons transfected with AAV-hSyn1-GCaMP6s-P2A-nls-dTomato, aligned to pinch stimulation in CPN mice, alongside representative calcium signal traces from a single animal (3 trials). **F** Representative fluorescence signals (Δ*F*/*F*) (the average Δ*F*/*F* response across 3 trials from a single mouse) of GCaMP6s (red) and control (blue) recorded from RMC^CaMKIIα^ neurons. **G** Quantification of Ca^2+^ signal in RMC neurons with fiber photometry (the statistical summary (mean ± SEM) of peak of Δ*F*/*F* across 3 trials for individual mice in each group) (*n *= 9 trials for Sham+GcaMP6s, *n *= 9 trials for CPN+GcaMP6s from 3 mice each group). **H** Schematic of electrophysiological recording from RMC in brain slices of mice after CPN. **I** The representative field potentials of RMC neurons in sham and CPN-ligated mice and the stimulation time points. **J** Representative action potentials of RMC neurons in sham and CPN-ligated mice. **K** Quantification of action potentials of RMC neurons as shown in (**I**) (*n *= 21 neurons for the sham group from 4 mice, *n *= 22 neurons for the CPN group from 4 mice). **L**, **M** Quantification of peak and decay time of field potentials of RMC neurons in sham and CPN-treated mice (*n* = 12 groups of neurons for the sham group from 5 mice, *n* = 13 groups of neurons for the CPN group from 3 mice). **N** Representative traces of mEPSC recordings in RMC neurons from sham and CPN-ligated mice. **O** Quantification graphs of frequency and amplitude of mEPSC in RMC neurons from sham and CPN-ligated mice (*n *= 20 neurons for the sham group from 3 mice, *n *= 41 neurons for the CPN group from 4 mice). **P** Cumulative probability plots of mEPSC frequency of RMC neurons from sham and CPN-ligated mice. Data are presented as the mean ± SEM, *t*-test or two-way ANOVA, ^*^P < 0.05, ^**^P < 0.01.

We first examined neuronal activity patterns in RMC of mice with neuropathic pain after CPN ligation (Fig. 1A). The percentage of c-Fos⁺ neurons (a marker of neuronal activation) in the RMC was significantly increased one week post-CPN (Fig. 1B). Most c-Fos⁺ neurons co-localized with CaMKIIα (Ca^2+^/calmodulin-dependent protein kinase IIα, a marker of glutamatergic neurons) (Fig. 1B), suggesting that CaMKIIα^+^ neurons in RMC were activated in male mice after CPN ligation. To exclude nonspecific background immunostaining of c-Fos, Fos-Cre^ERT2^; Ai14 transgenic male mice were employed to specifically tag cells activated by CPN. This transgenic line enables Cre-dependent tdTomato expression in c-Fos-expressing neurons following treatment with 4-OHT [23, 24]. Interestingly, as shown in Fig. 1C, the percentage of tdTomato^+^ neurons was significantly increased in mice after CPN ligation, particularly in RMC, which was consistent with c-Fos immunostaining results. Taken together, these results strongly suggested that CaMKIIα^+^ neurons in RN, especially RMC (RMC^CaMKIIα^), were activated in male mice with neuropathic pain after CPN ligation.

To further explore the dynamic activity of RMC^CaMKIIα^ neurons in mice with neuropathic pain-like hypersensitivity, we monitored Ca^2^⁺ levels (an indicator of neuronal activity) using *in vivo* fiber photometry. AAV-hSyn1-GCaMP6s- P2A-nls-dTomato viruses (a genetically encoded fluorescent Ca^2+^ indicator) were injected into RMC (Fig. 1D). As expected, a noxious pinch stimulus induced significant Ca^2^⁺ elevation in the RMC post-CPN (Fig. 1E–G), indicating RMC neurons were activated in these mice. Subsequently, we examined the electrophysiological properties of these RMC neurons in CPN-ligated mice, including field potentials, action potentials, and mEPSCs. Indeed, as shown in Fig. 1H, I, field potentials in brain slices were reliably evoked in male mice after CPN ligation. The firing rates of action potentials in RMC neurons were significantly increased in mice after CPN ligation (Fig. 1J–M). Additionally, the frequency and amplitude of mEPSCs in RMC neurons from brain slices of CPN-ligated mice were significantly increased (Fig. 1N–P). Taken together, these results strongly suggested that RMC^CaMKIIα^ neurons were activated in male mice with neuropathic pain after CPN ligation.

## Inhibition or Induced Apoptosis of CPN-activated Neurons in RMC Alleviated the Neuropathic Pain in Male Mice After CPN Ligation

**Fig. 2:**
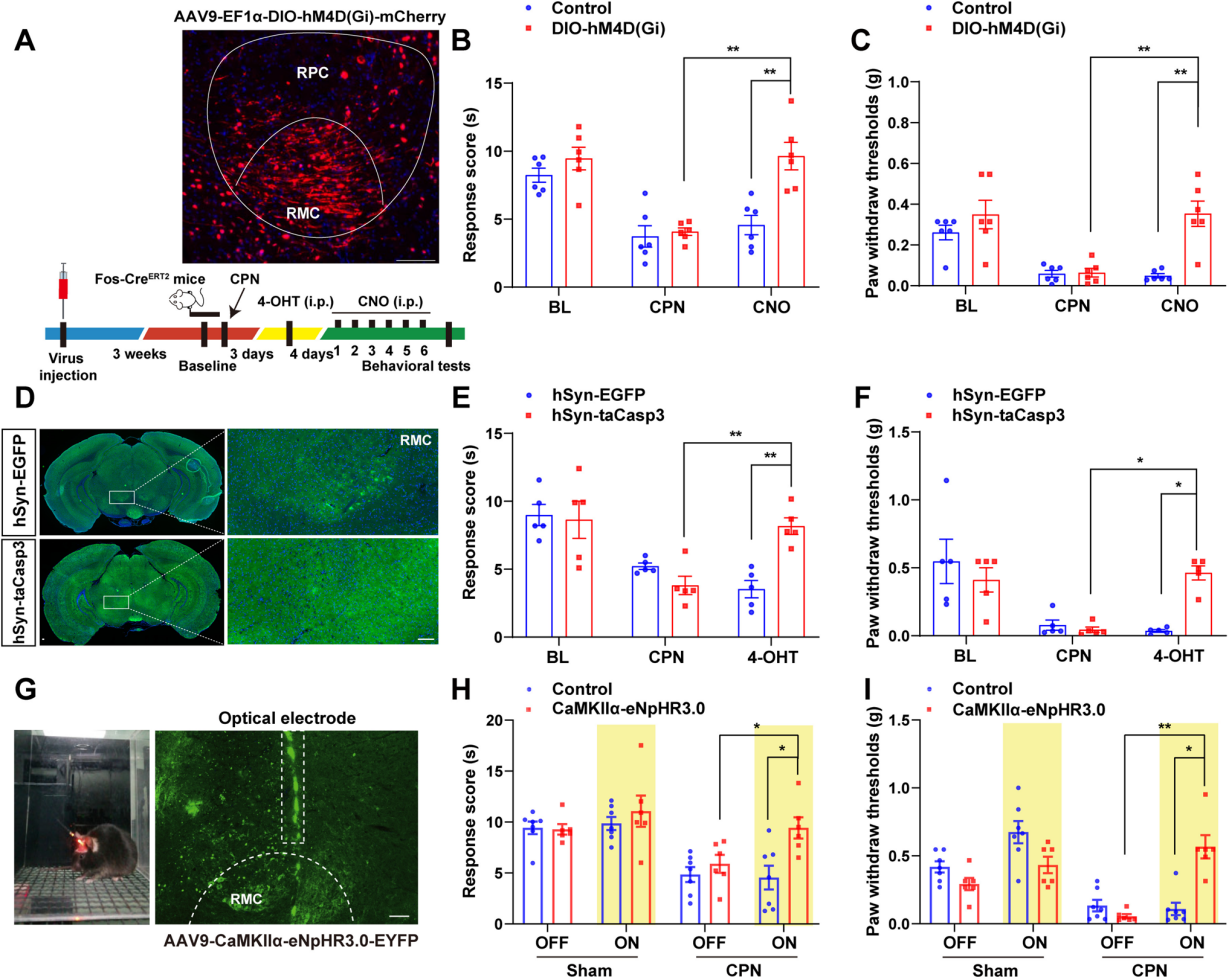
Inhibition or induced apoptosis of CPN-activated neurons in RMC alleviated the neuropathic pain in male mice after CPN ligation. **A** Representative photomicrograph of the viral injection site in RMC and the behavioral experiments design. Scale bar, 100 μm. **B** Quantification of thermal response score in mice expressing Control or AAV9-EF1α-DIO-hM4D(Gi)-mCherry (DIO-hM4D(Gi)) viruses after CPN ligation (*n *= 6 mice per group). **C** Quantification of paw withdraw thresholds in mice expressing Control or DIO-hM4D(Gi) viruses after CPN ligation (*n* = 6 mice per group). **D** Representative images of RMC neurons transfected with Control or AAV-hSyn-taCasp3-T2A-TEVp-P2A-EGFP-3xFLAG-WPRE (hSyn-taCasp3) viruses. Scale bar, 100 μm. **E** Quantification of thermal response score in mice transfected with Control or AAV-hSyn-taCasp3-T2A-TEVp-P2A-EGFP-3xFLAG-WPRE (hSyn-taCasp3) viruses after CPN ligation (*n *= 5 mice per group). **F** Quantification of paw withdraw thresholds in mice transfected with Control or hSyn-taCasp3 viruses after CPN ligation (*n *= 5 mice per group). **G** Schematic of light illumination and virus injection for expression of AAV9-CaMKIIα-eNpHR3.0-EYFP (CaMKIIα-eNpHR3.0) in RMC. Scale bar, 100 μm. **H** Quantification of thermal response score in mice transfected with Control or CaMKIIα-eNpHR3.0 viruses after CPN ligation (*n *= 7 mice for Control group, *n *= 6 mice for CaMKIIα-eNpHR3.0 group). **I** Quantification of paw withdraw thresholds in mice transfected with Control or CaMKIIα-eNpHR3.0 after CPN ligation (*n *= 7 mice for the Control group, *n *= 6 mice for the CaMKIIα-eNpHR3.0 group). Data are presented as the mean ± SEM, two-way repeated measures (RM) ANOVA, ^*^P < 0*.*05*,* ^**^P < 0.01.

To further examine the roles of RMC neurons in neuropathic pain, AAV9-EF1α-DIO-hM4D(Gi)-mCherry viruses were injected bilaterally into RMC in Fos-Cre^ERT2^ mice to selectively silence the activity of activated RMC neurons (Fig. 2A). Three weeks post-infection, CPN ligation was performed. One week later, the hM4D(Gi) agonist CNO was administered i.p. once daily for 6 days (Fig. 2A). Thermal response latency (Hargreaves test) and mechanical allodynia threshold (Von Frey test) were assessed. As expected, both thermal hyperalgesia (Fig. 2B) and mechanical allodynia (Fig. 2C) were alleviated in mice expressing hM4D(Gi)-mCherry but not the control group after CNO injection. Previous studies have reported that RN is involved in the modulation of motor control [25, 26]. To rule out the potential effects on pain thresholds by movement and to determine whether RN was involved in regulating anxiety-like behaviors caused by pain, open field and elevated plus maze tests were conducted in these mice. The behavioral test results showed normal locomotion and emotional responses in these mice (Fig. S1A–E). Although chronic pain and anxiety were often comorbid, probably due to the changes of long-term plasticity in some brain structures, our results suggested that short-term stimulation of activated RMC neurons did not induce anxiety-like behaviors in mice. Collectively, these results suggested that chemogenetic inhibition of CPN-activated neurons in RMC alleviated the neuropathic pain in mice after CPN.

To further confirm the involvement of CPN-activated neurons in RMC in pain modulation, AAV-hSyn-taCasp3-T2A-TEVp-P2A-EGFP-3xFLAG-WPRE viruses were then injected bilaterally into the RMC of Fos-Cre^ERT2^ mice to selectively induce apoptosis of activated neurons by CPN. As shown in Fig. 2D, c-Fos^+^ neurons were indeed induced to apoptosis. Furthermore, we found that neuropathic pain was alleviated in these mice after the induction of 4-OHT (Fig. 2E, F); however, the locomotor activity was not significantly affected in these mice (Fig. S1F–J). Taken together, these results suggested that elimination of CPN-activated neurons in RMC alleviated the neuropathic pain in mice after CPN.

To further elucidate the roles of RMC^CaMKIIα^ neurons in neuropathic pain, the optogenetic inhibition experiments were performed. RMC^CaMKIIα^ neurons in CPN-ligated mice were bilaterally infected with AAV9-CaMKIIα-eNpHR3.0-EYFP, expressing halorhodopsin to silence neuronal firing *via* hyperpolarization upon yellow light [27–29]. Virus infections were mostly restricted to RMC without leakage into other brain regions (Fig. 2G). As expected, illumination of RMC^CaMKIIα^ neurons expressing eNpHR3.0-EYFP but not control viruses also alleviated both thermal hyperalgesia (Fig. 2H) and mechanical allodynia (Fig. 2I) in these mice after CPN. Meanwhile, we also confirmed that the optogenetic inhibition of RMC^CaMKIIα^ neurons did not induce anxiety-like behaviors (Fig. S1K–O). Taken together, these results suggested that chemogenetic or optogenetic inhibition or induced apoptosis of RMC^CaMKIIα^ neurons alleviated the neuropathic pain in mice after CPN ligation.

## Activation of RMC^CaMKIIα^ Neurons Sufficiently Induced the Thermal Hyperalgesia and Mechanical Allodynia in Male Naïve Mice

**Fig. 3:**
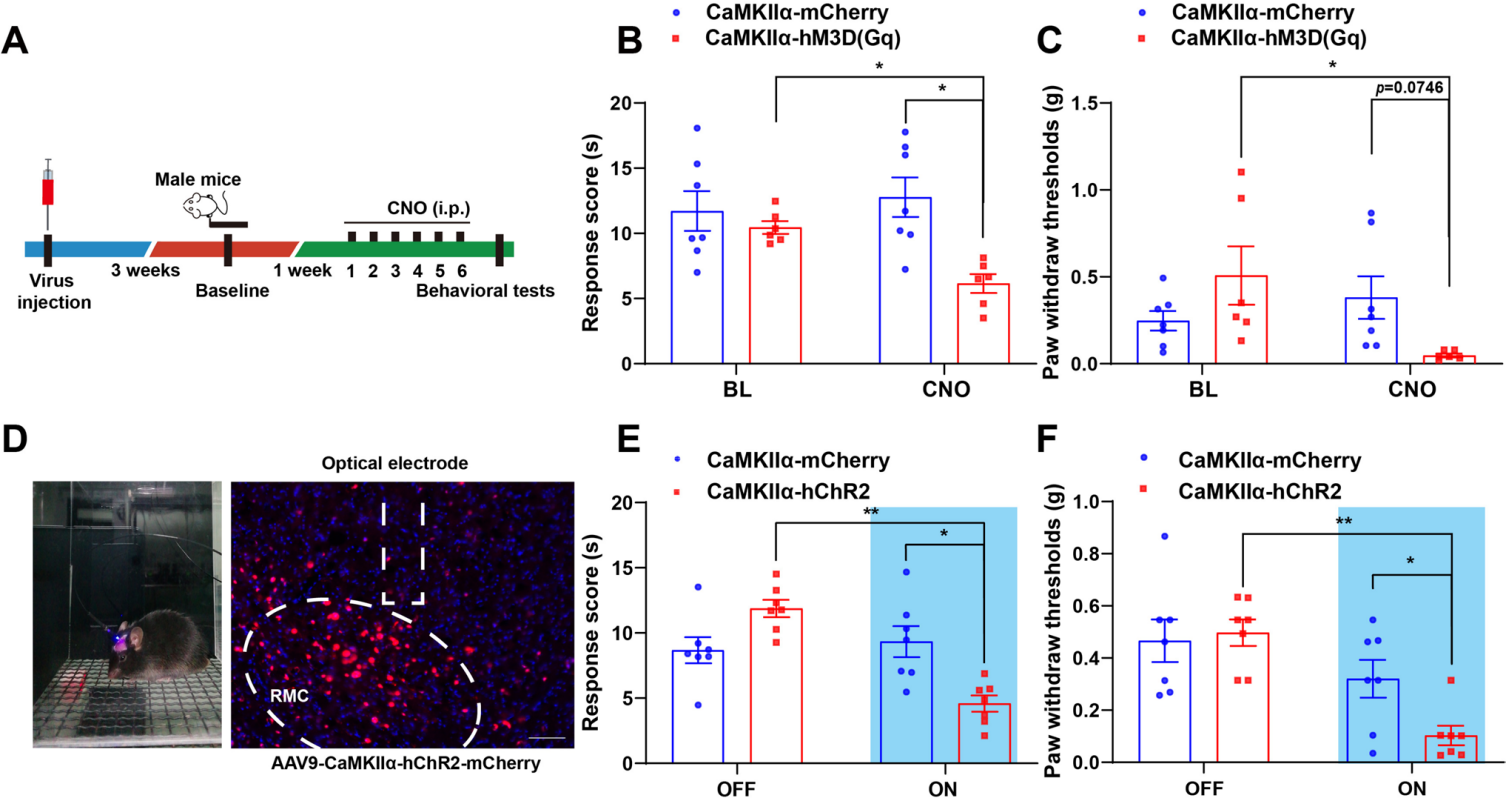
Activation of RMC^CaMKIIα^ neurons sufficiently induced the mechanical allodynia and thermal hyperalgesia in naïve male mice. **A** Experimental design and timeline of behavioral experiments. **B** Quantification of the thermal stimulus response latency for AAV9-CaMKIIα-mCherry (CaMKIIα-mCherry) or AAV9-CaMKIIα-hM3D(Gq)-mCherry (CaMKIIα-hM3D(Gq)) infected mice in the Hargreaves test (*n *= 7 mice for CaMKIIα-mCherry group, *n *= 6 mice for CaMKIIα-hM3D(Gq) group). **C** Quantification of paw withdraw thresholds for CaMKIIα-mCherry or CaMKIIα-hM3D(Gq) infected mice in Von Frey filament test (*n *= 7 mice for CaMKIIα-mCherry group, *n *= 6 mice for CaMKIIα-hM3D(Gq) group). **D** Schematic of light illumination and virus injection for expression of AAV9-CaMKIIα-hChR2-mCherry (CaMKIIα-hChR2) in RMC. Scale bar, 100 μm. **E** Quantification of thermal stimulus response latency upon RMC-optogenetic stimulation between CaMKIIα-mCherry and CaMKIIα-hChR2 groups (*n *= 7 mice per group). **F** Quantification of paw withdraw thresholds upon RMC-optogenetic stimulation between CaMKIIα-mCherry and CaMKIIα-hChR2 groups (*n *= 7 mice per group). Data are presented as the mean ± SEM, two-way RM ANOVA, ^*^P < 0.05, ^**^P < 0.01.

We next explored whether activation of RMC^CaMKIIα^ neurons sufficiently induced allodynia and hyperalgesia, hallmarks of neuropathic pain in mice. AAV9-CaMKIIα-hM3D(Gq)-mCherry viruses were microinjected bilaterally into RMC to activate excitatory neurons in male naïve mice (Fig. 3A). As expected, chemogenetic activation of RMC^CaMKIIα^ neurons expressing hM3D(Gq)-mCherry induced significant thermal hyperalgesia (Fig. 3B). Meanwhile, mechanical pain thresholds showed a downward trend following CNO administration (*P* = 0.0746) (Fig. 3C), as evidenced by reduced withdrawal thresholds to heat stimuli and von Frey filaments, respectively. Moreover, we also assessed the locomotor activity of these mice after CNO injection (Fig. S2A). There were no significant differences in the total distance traveled and time spent in the center assessed by open field assays (Fig. S2B, C), nor in the duration and number of entries into open arms assessed by elevated plus maze in these mice (Fig. S2D, E). These results suggested that locomotor activity was not affected and anxiety-like behaviors were not induced in these mice by chemogenetic manipulation.

To further confirm the roles of RMC^CaMKIIα^ neuron activation in neuropathic pain, we next employed an optogenetic approach. Optical fibers were implanted into the RMC of mice pre-injected with AAV9-CaMKIIα-hChR2-mCherry viruses (Fig. 3D). Optogenetic manipulation of these neurons resulted in thermal hyperalgesia (Fig. 3E) and mechanical allodynia (Fig. 3F) in these treated mice compared to their pre-illumination baseline, in contrast to controls that showed no threshold alterations. Further analysis of movement effects (Fig. S2F) showed there were no significant differences between optogenetics-treated and control mice in terms of total distance traveled or central zone entries in open field assays (Fig. S2G, H), nor in time and entries into open arms of elevated plus maze (Fig. S2I, J). Taken together, these results suggested that chemogenetic activation of RMC neurons significantly induced thermal hyperalgesia, but produced a trend towards reduced mechanical pain thresholds. In contrast, optogenetic activation of RMC^CaMKIIα^ neurons was sufficient to elicit both mechanical allodynia and thermal hyperalgesia in naïve male mice, without impairing motor function.

## Long-range Projections from RMC to DH Facilitated the Neuropathic Pain in Male Mice After CPN Ligation

**Fig. 4:**
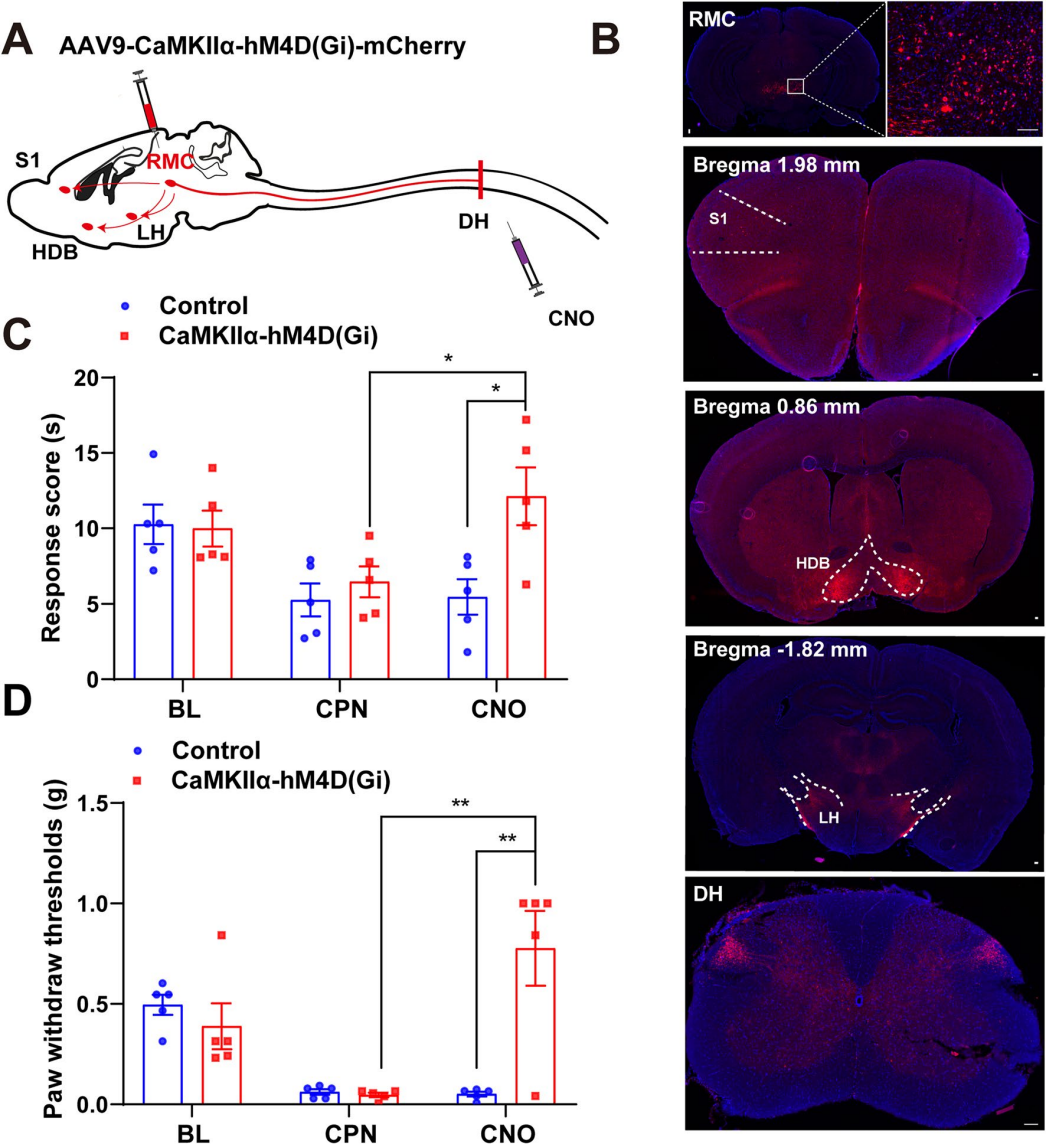
Long-range projections from RMC to DH neurons facilitated the neuropathic pain in male mice after CPN ligation. **A** Schematic of the virus and CNO injection. **B** Representative micrographs showed the injection site within RMC and projection of RMC neurons to the primary somatosensory cortex (S1), horizontal limb of the diagonal band (HDB), lateral hypothalamus (LH), and DH. Scale bars, 100 μm. **C, D** Quantification of response score (**C**) and paw withdraw thresholds (**D**) in mice expressing Control or AAV9-CaMKIIα-hM4D(Gi)-mCherry (CaMKIIα-hM4D(Gi)) viruses after CPN ligation in Hargreaves test and Von Frey filament test (*n *= 5 mice per group). Data are presented as the mean ± SEM, two-way RM ANOVA, ^*^P < 0*.*05*,* ^**^P < 0*.*01.

To explore the neural circuitry underlying RMC’s pain modulation, AAV9-CaMKIIα-hM4D(Gi)-mCherry viruses were injected into RMC bilaterally to trace mCherry-labeled CaMKIIα^+^ neurons and their fiber projections. Interestingly, as shown in Fig. 4A, B, RMC^CaMKIIα^ neurons were found to project to several key regions, including primary somatosensory cortex (S1), horizontal limb of the diagonal band (HDB), lateral hypothalamus (LH) and spinal dorsal horn (DH) [30–32], we next investigated function of RMC-DH neural circuit in the regulation of neuropathic pain. Intrathecal CNO injections were used to inhibit the activity of RMC-projecting neurons expressing hM4D(Gi)-mCherry in the spinal cord. Interestingly, both thermal hyperalgesia (Fig. 4C) and mechanical allodynia (Fig. 4D) were alleviated in mice expressing AAV9-CaMKIIα-hM4D(Gi)-mCherry but not in those expressing the control virus after CNO injection. However, the locomotor activity was not significantly affected in these mice (Fig. S3A–E). These results suggested that the RMC-DH CaMKIIα^+^ neural circuit mediated the facilitation of neuropathic pain in male mice after CPN ligation, as demonstrated by pain relief following its chemogenetic inhibition.

## RMC^CaMKIIα^-DH^CaMKIIα^ Circuit Facilitated the Neuropathic Pain in Male Mice After CPN Ligation

**Fig. 5:**
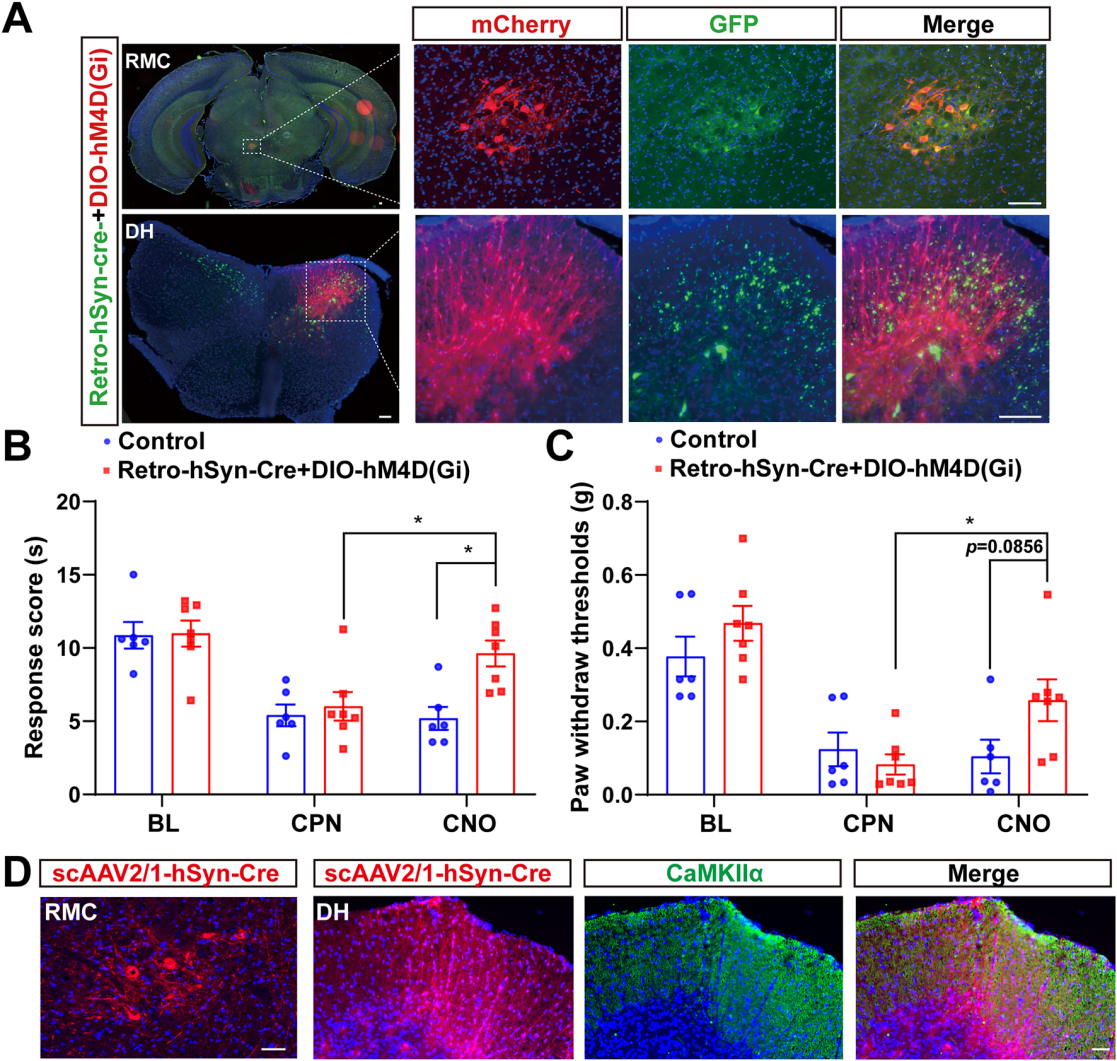
RMC^CaMKIIα^-DH^CaMKIIα^ neural circuit facilitated the neuropathic pain in male mice after CPN ligation. **A** Representative images showing neurons in the dorsal horn (DH) that were microinjected with AAV2-retro-hSyn-Cre-P2A-GFP, exhibiting co-localization with AAV9-EF1α-DIO- hM4D(Gi)-mCherry (Retro-hSyn-Cre+DIO-hM4D(Gi)) viruses in RMC. Scale bars, 100 μm. **B, C** Quantification of thermal paw-withdraw latency (**B**) and PWT (**C**) in CPN-ligated mice upon RMC-DH circuit chemo-inhibition (*n *= 6 mice for the Control group, *n *= 7 mice for the Retro-hSyn-Cre+DIO-hM4D(Gi) group). **D** Representative image showing neurons in the DH that were transduced by scAAV2/1-hSyn-Cre-pA injected into the RMC of an Ai14 mouse and were co-localized with the excitatory neuronal marker CaMKIIα. Scale bars, 50 μm. Data were mean ± SEM, two-way RM ANOVA, ^*^P < 0.05.

To further elucidate the roles of the RMC-DH circuit in the regulation of neuropathic pain, Cre-dependent selection yields AAV variants AAV2-retro-hSyn-Cre-P2A-GFP viruses were injected into DH, and subsequently AAV9-EF1α-DIO-hM4D(Gi)-mCherry viruses were injected into RMC after one week. AAV2-retro, a new capsid variant capable of efficient retrograde transport, was injected into the target region of interest and labelled only neurons in other locations of the nervous system that extend their axons to the injected target region [33]. AAV2-retro viruses can mediate the expression of Cre recombinase to form a synapse at the injection site. This strategy can lead to a knockout of floxed alleles or activation of a floxed reporter [34]. Interestingly, retrograde tracing analysis results showed that the projections of DH neurons indeed retrograded to RMC neurons (Fig. 5A). Chemogenetic inhibition of RMC-DH circuits significantly alleviated thermal hyperalgesia (Fig. 5B) and tended to reduce mechanical allodynia in mice with CPN ligation (*P =*0.0856) (Fig. 5C), without affecting locomotor function or provoking emotional responses (Fig. S3F–J). Utilizing anterograde monosynaptic viruses (scAAV2/1-hSyn-Cre-pA) tracing, we found that DH neurons receiving RMC projections were CaMKIIα^+^ (DH^CaMKIIα^) neurons (Fig. 5D). These results, together with the above results, suggested that DH neurons received the innervation from local RMC^CaMKIIα^ neurons, and the RMC^CaMKIIα^-DH^CaMKIIα^ circuit facilitated neuropathic pain in male mice after CPN ligation.

**Fig. 6:**
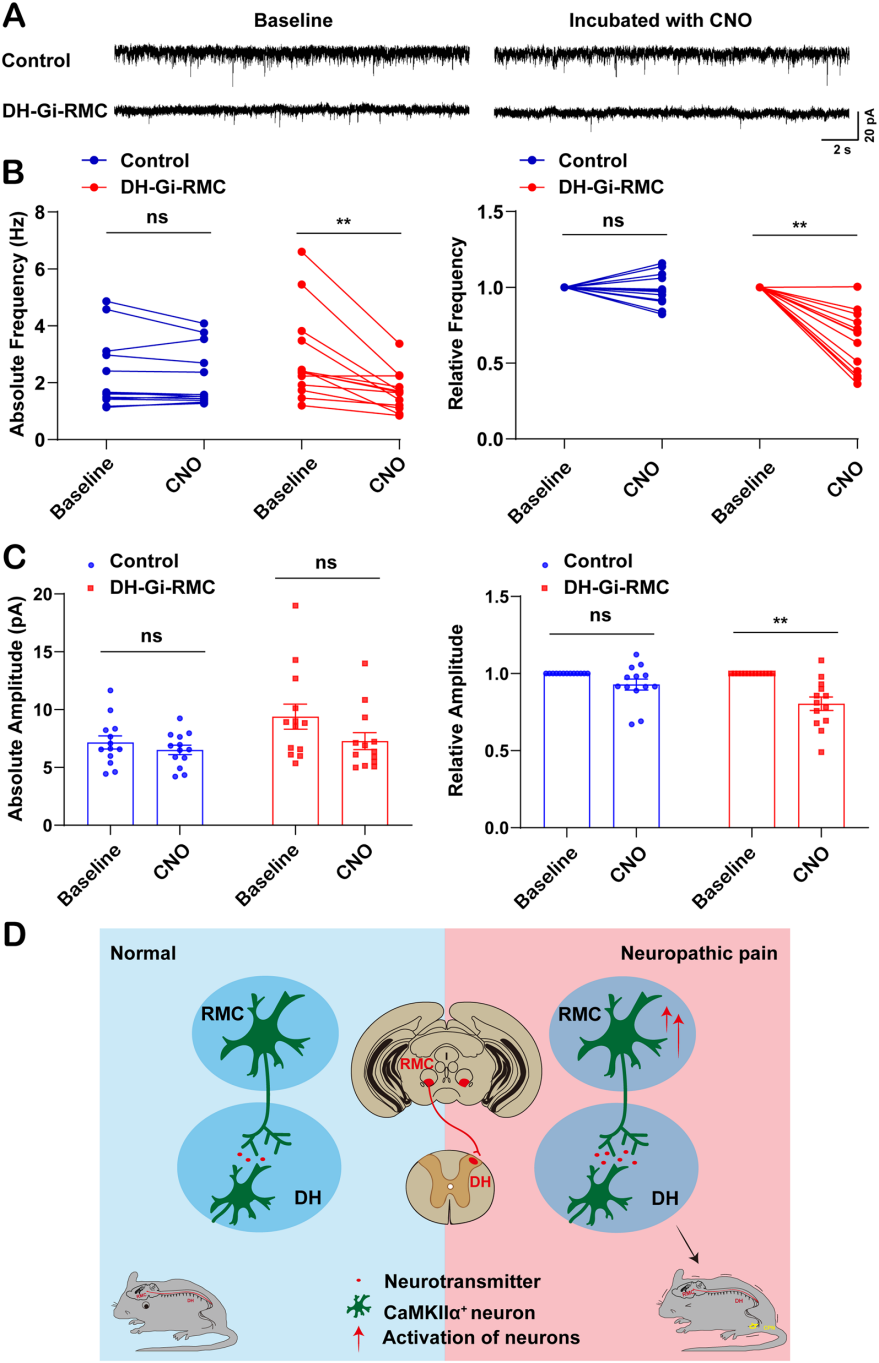
Electrophysiological properties of RMC^CaMKIIα^-DH^CaMKIIα^ circuit in CPN-ligated mice and working model of RMC^CaMKIIα^-DH^CaMKIIα^ circuit in the regulation of neuropathic pain in male mice. **A** Representative traces of mEPSCs recordings in RMC neurons from male CPN-ligated mice receiving dual viral injections [AAV2-retro-hSyn-Cre-P2A-GFP in the DH and AAV9-EF1α-DIO-hM4D(Gi)-mCherry in the RMC (DH-Gi-RMC)]. **B** Quantification of absolute and relative frequency of mEPSC in RMC neurons from male CPN-ligated mice receiving dual viral injections (*n *= 13 neurons per group). **C** Quantification of absolute and relative amplitude of mEPSC in RMC neurons from male CPN-ligated mice receiving dual viral injections (*n *= 13 neurons per group). **D** Working model of RMC^CaMKIIα^-DH^CaMKIIα^ circuit in the regulation of neuropathic pain in male mice. In this model, RMC^CaMKIIα^ neurons are activated in male mice after CPN and project to DH^CaMKIIα^ neurons. A novel long-range projection of RMC^CaMKIIα^ to DH^CaMKIIα^ neural circuit is identified to control the development and maintenance of neuropathic pain in male mice. Red arrows indicate the increase in activation of RMC^CaMKIIα^ neurons. Data are presented as the mean ± SEM, two-way ANOVA, ^**^P < 0*.*01, ns, no significance.

Additionally, we also performed the electrophysiological recordings on neurons of RMC retrograde traced from DH during chemogenetic activation of hM4D(Gi). We found that the firing rate of RMC neurons was significantly decreased after chemogenetic inhibition, as illustrated by mEPSC traces (Fig. 6A). The activity of hM4D(Gi)-expressing dorsal-RMC neurons was significantly suppressed after CNO treatment (Fig. 6A). Both mEPSC frequency and relative amplitude were significantly reduced (Fig. 6B, C), altering the absolute amplitude in male mice transfected with AAV2-retro-hSyn-Cre-P2A-GFP in DH and AAV9-CaMKIIα-hM4D(Gi)-mCherry in RMC (DH-Gi-RMC), compared with that in the control mice after CPN ligation. Taken together, these results strongly suggested that inhibition of the RMC^CaMKIIα^-DH^CaMKIIα^ circuits alleviated neuropathic pain, indicating that this circuit underlied the facilitation of pain in male mice after CPN ligation.

## Discussion

In this study, we provide evidence that RMC^CaMKIIα^ neurons are activated in male mice with neuropathic pain and reveal a novel neural circuit from RMC to DH that facilitates neuropathic pain in male mice. Chemogenetic or optogenetic inhibition of these RMC^CaMKIIα^ neurons alleviates neuropathic pain in male mice after CPN, while their activation induces the neuropathic pain-like behaviors in male naïve mice. Finally, a novel long-range projection of RMC^CaMKIIα^-DH^CaMKIIα^ neural circuit is identified to facilitate the neuropathic pain in male mice, which will potentially be exploited for targeting neuropathic pain (Fig. 6D).

Initially, many studies have shown that RN primarily links motor areas of the cerebral cortex with the cerebellum and spinal cord, and contributes to voluntary movements [25, 35–37]. In recent years, a few findings in rat models and human neuroimaging imply a role for RN in mediating antinociceptive responses to pain stimulation [38]. As demonstrated in our experiment (Fig. S1M), optogenetic activation of the RMC resulted in an increase in the total distance traveled by the mice. We speculate that the observed hyperlocomotion may have been partly attributable to unintended viral spread to the adjacent RPC, leading to its concomitant activation. Nonetheless, the pronociceptive effects we observed under RMC activation remain robust and functionally distinct, supporting the conclusion that RMC modulates pain responses independently of its potential role in motor enhancement. The significant difference observed in Fig. S3E is potentially attributable to the role of the spinal dorsal horn in regions associated with anxiety regulation. However, the precise underlying mechanism warrants further investigation. Fox *et al*. have demonstrated that the RN is activated by purely sensory stimuli in the absence of the opportunity to coordinate finger movements or to use the sensory cues to guide movement [39]. Zeng *et al*. have reported that RN-derived IL-6 mediates the maintenance of neuropathic pain by inducing the production of tumor necrosis factor-alpha (TNF-α) and interleukin-1*β* (IL-1*β*) [17]. These precise neural circuits and temporal dynamics underlying RN involvement in neuropathic pain pathogenesis remain poorly defined. Our study advances this field through four key innovations: First, we demonstrated the functional recruitment of CaMKIIα^+^ neurons, particularly within the RMC, in response to noxious stimuli. Second, by integrating Fos-Cre^ERT2^ mice with chemogenetic strategies, we established a cell-type-specific targeting approach for CPN-activated RMC neurons, significantly enhancing spatial and functional precision beyond prior methodologies. Third, our electrophysiological analyses revealed comprehensive neuronal hyperexcitability in CPN-treated mice, evidenced by increased field potentials, action potentials, and mEPSC amplitude in RMC neurons (Fig. 1H–P). Fourth, we identified and functionally validated a previously unrecognized RMC-DH circuit as a critical pathway mediating neuropathic pain persistence in male mice after CPN ligation. Through the combination of anterograde and retrograde tracing, *in vivo* chemogenetic inhibition, and specific electrophysiological recording, we demonstrated that RMC neurons projected directly to the DH to facilitate the neuropathic pain in male mice after CPN ligation (Figs. 4–6). Collectively, these findings provide unprecedented circuit-level insights into RMC-mediated neuropathic pain mechanisms in male mice.

Cytoarchitecturally, RN is differentiated into RMC and RPC. Phylogenetic studies indicate a progressive divergence within RN, with the magnocellular component associated with the rubrospinal system and parvocellular component linked to the olivocerebellar system [40–42]. The large majority of RMC neurons (91.5%) responded to noxious stimulation of skin in the cat [43]; however, RPC may provide with recurrent loop returning *via* the thalamus to the motor cortex [44]. Our studies identified that RMC^CaMKIIα^ neurons projected to the spinal dorsal horn to regulate neuropathic pain (Figs 4B and 5A). The spinal dorsal horn has been regarded as the entry point into the central nervous system for somatosensory information from trunk and limbs [45]. Altered function of its related circuits contributes to chronic pain [46–48]. Our electrophysiology and behavioral analyses revealed that inhibition of the RMC-DH circuit decreased the firing rate of RMC neurons and alleviated neuropathic pain-like hypersensitivity (Figs. 4–6). Diverging from established models of rubrospinal function, our study identifies a previously unrecognized noncanonical excitatory projection from the RMC to the DH as a dedicated circuit driver of neuropathic pain pathogenesis.

In the central nervous system, sensory information is afferent from the peripheral nerve sensory endings to the spinal cord and transmitted further upward to the sensory cortex. But motor information originates from the motor cortex and is transmitted directly to the spinal cord [49–51]. Previous studies have shown that ACC directly potentiates spinal sensory transmission [52]. Consistent with previous results [53, 54], we found that RMC projected to the spinal cord to regulate neuropathic pain. After peripheral nerve injuries, the neuronal activity in RMC was potentiated, and the release of glutamate was increased. It revealed that RMC-DH output neurons release glutamate as a transmitter. The persistently activated RMC neurons will cause a tonic potentiation effect on the spinal sensory transmission and contribute to the maintenance of behavioral hyperalgesia. Given that the RN is rich in blood vessels (this is also the reason why it is called the Red nucleus), it can provide a sustained supply of energy for the activation of excitatory neurons [35, 55]. We hypothesize that the activation of these excitatory neurons in the RMC can continuously potentiate excitatory neurons in the spinal dorsal horn. This, in turn, augments the excitatory input from the dorsal horn neurons to other pain-regulating regions in the central nervous system. Ultimately, this cascade of events leads to central sensitization and the maintenance of chronic pain (Fig. 6D). However, the role of RMC-DH in the development of neuropathic pain needs to be further elaborated. Chronic pain following spinal cord injury (SCI) is common and may result in a substantially reduced quality of life [56, 57]. It is possible that robust nociceptive signaling from the SCI may activate the RMC-DH circuit and lead to the development of neuropathic pain-like hypersensitivity. It is worth testing the RMC-DH circuit, which may be one of the underlying pathophysiological mechanisms of neuropathic pain after SCI in the future [58].

Neuropathic pain continues to pose a significant public health challenge, with effective treatments remaining scarce. Our results posit the RMC as a promising target for therapeutic intervention in neuropathic pain. Focusing on neural circuits of RMC, a relatively novel brain region in chronic pain, our findings may pave the way for innovative treatment approaches and pharmaceutical development for neuropathic pain.

## Supplementary Information

Below is the link to the electronic supplementary material.

Supplementary file1 (PDF 1019 KB)

## Data Availability

Data related to this study are available upon reasonable request.
