# Supplementary material for: A Novel Long-range Excitatory Neural Circuit from the Magnocellular Red Nucleus to Spinal Dorsal Horn Facilitates Neuropathic Pain-like Behaviors in Male Mice
Source: Neurosci Bull. 2025 Dec 24;42(6):1311–24. doi: 10.1007/s12264-025-01553-7 (PMC13221545; doi:10.1007/s12264-025-01553-7)
Supplement: Supplementary file 1 — Supplementary file1 (PDF 1019 KB) [file 12264_2025_1553_MOESM1_ESM.pdf]

Supplementary Materials

Supplementary Figures

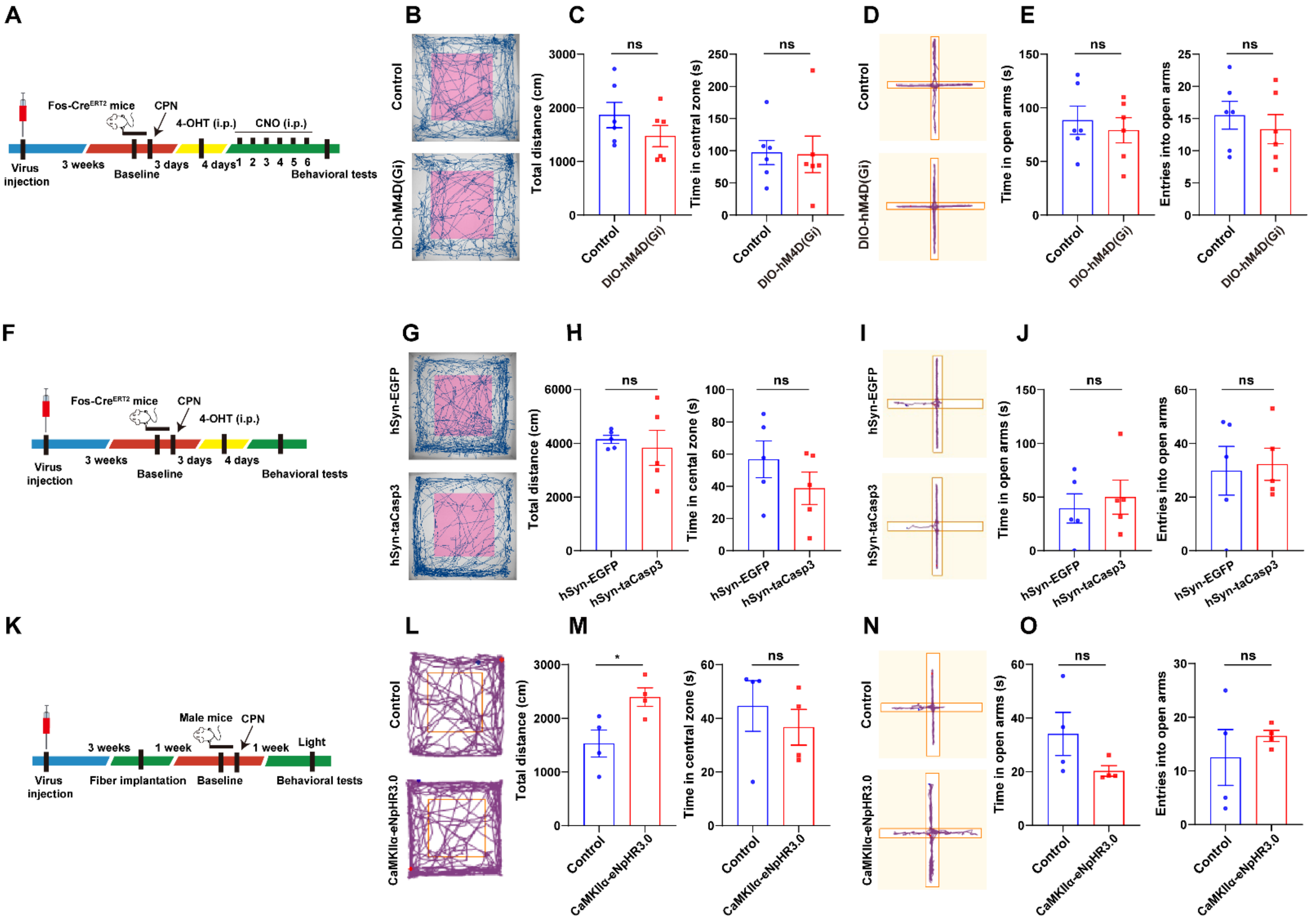

Fig. S1 Chemogenetic or optogenetic inhibition of RMC^{CaMKIIα} neurons did not affect locomotion and did not induce anxiety-like behaviors in male mice after CPN ligation. **A** The experimental design and timeline for behavioral testing following chemogenetic inhibition of RMC^{CaMKIIα} neurons. **B** Representative tracks of mice transfected with viruses in the open field test after CPN ligation. **C** Quantification of total distance moved and time spent in the center in the open field test (*n* = 6 mice per group). **D** Representative tracks of mice transfected with viruses in the elevated plus maze test after CPN ligation. **E** Quantification of time in open arms and entries into open arms in the elevated plus maze test (*n* = 6 mice per group). **F** The experimental design and timeline for behavioral testing. **G** Representative tracks of mice transfected with Control or hSyn-taCasp3 viruses in the open field test after CPN ligation. **H** Quantification of total distance moved and time spent in the center in the open field test (*n* = 5 mice per group). **I** Representative tracks of mice transfected with Control or hSyn-taCasp3 viruses in the elevated plus maze test after CPN ligation. **J** Quantification of time in open arms and entries into open arms in the elevated plus maze test (*n* = 5 mice per group). **K** The

experimental design and timeline for behavioral testing following optogenetic inhibition. **L** Representative tracks of mice transfected with Control or CaMKIIα-eNpHR3.0 viruses in the open field test after CPN ligation. **M** Quantification of total distance moved and time spent in the center in the open field test (*n* = 4 mice per group). **N** Representative tracks of mice transfected with Control or CaMKIIα-eNpHR3.0 in the elevated plus maze test after CPN ligation. **O** Quantification of time in open arms and entries into open arms in the elevated plus maze test (*n* = 4 mice per group). Data are presented as the mean ± SEM, *t*-test, $^{*}P < 0.05$, ns, no significance.

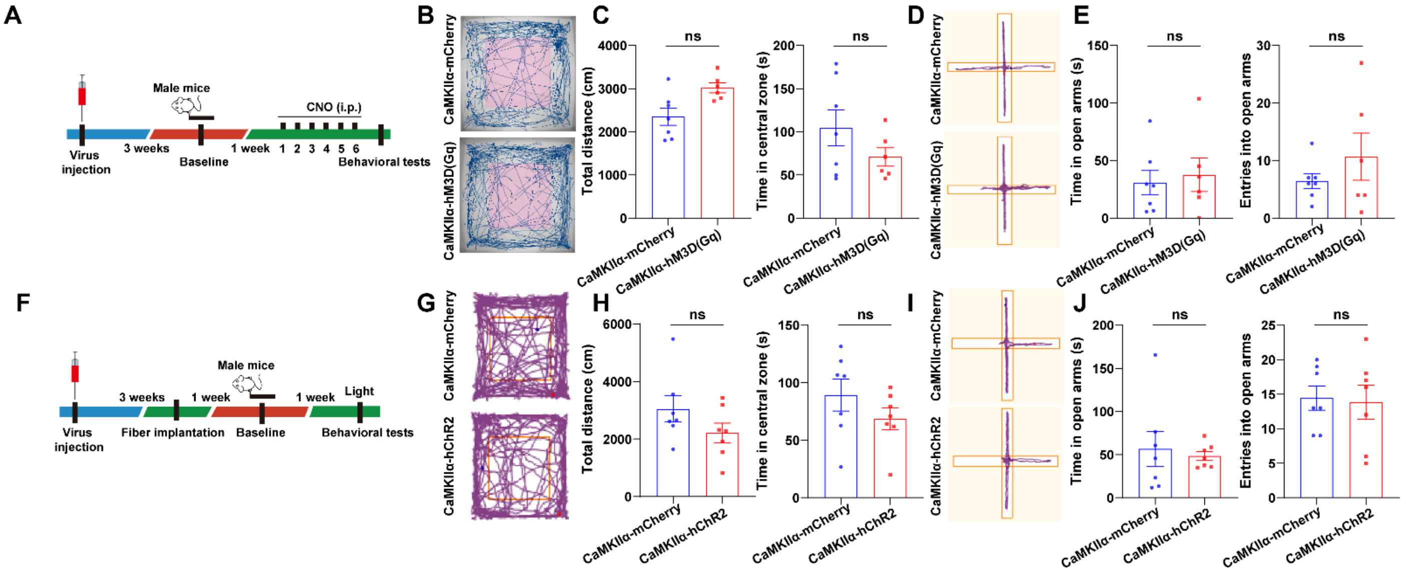

Fig. S2 Chemogenetic or optogenetic activation of RMC$^{CaMKII\alpha}$ neurons did not affect locomotion and did not induce anxiety-like behaviors in male mice after CPN ligation. **A** Experimental design and timeline for behavioral testing post-chemogenetic activation. **B** Representative tracks of mice transfected with CaMKIIα-mCherry or CaMKIIα-hM3D(Gq) viruses in the open field test. **C** Quantification of total distance moved and time spent in the center in the open field test (*n* = 7 mice for CaMKIIα-mCherry group, *n* = 6 mice for CaMKIIα-hM3D(Gq) group). **D** Representative tracks of mice transfected with CaMKIIα-mCherry or CaMKIIα-hM3D(Gq) viruses in the elevated plus maze test. **E** Quantification of time in open arms and entries into open arms in the elevated plus maze test (*n* = 7 mice for CaMKIIα-mCherry group, *n* = 6 mice for CaMKIIα-hM3D(Gq) group). **F** Experimental design and timeline for behavioral testing post-optogenetic activation. **G** Representative tracks of mice transfected with CaMKIIα-mCherry or CaMKIIα-hChR2 in the open field test. **H** Quantification of total distance moved and time spent in the center in the open field test (*n* = 7 mice per group). **I** Representative tracks of mice transfected with CaMKIIα-mCherry or CaMKIIα-hChR2 viruses in the elevated plus maze test. **J** Quantification of time in open arms and entries into open arms in the elevated plus maze test (*n* = 7 mice per group). Data are presented as the mean ± SEM, *t*-test, ns, no significance.

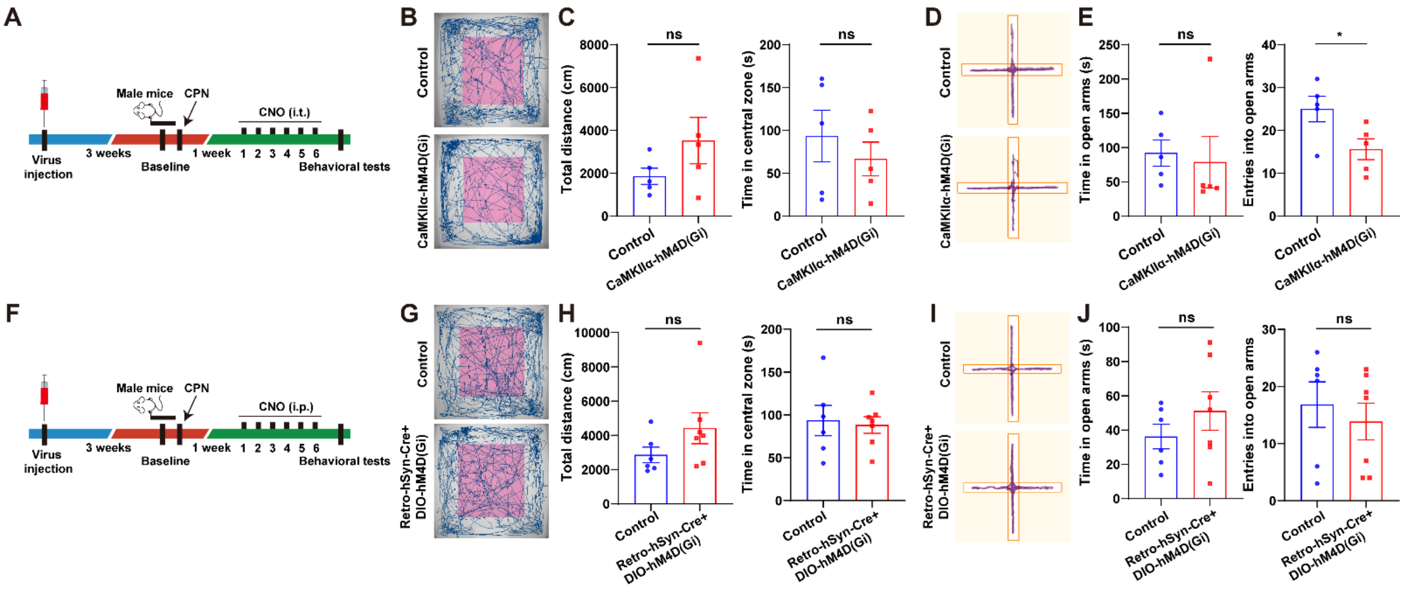

Fig. S3 Inhibition of RMC$^{CaMKII\alpha}$-DH$^{CaMKII\alpha}$ circuit did not affect locomotor activity and did not induce anxiety-like behaviors in male mice after CPN ligation. **A** The experimental design and timeline for behavioral testing following inhibition of the RMC$^{CaMKII\alpha}$-DH$^{CaMKII\alpha}$ circuit. **B** Representative tracks of mice transfected with Control or CaMKIIα-hM4D(Gi) viruses in the open field test, and CNO was injected intraperitoneally after CPN ligation. **C** Quantification of total distance moved and time spent in the center in the open field test (n = 5 mice per group). **D** Representative tracks of mice transfected with Control or CaMKIIα-hM4D(Gi) viruses in the elevated plus maze test after CPN ligation. **E** Quantification of time in open arms and entries into open arms in the elevated plus maze test (n = 5 mice per group). **F** The experimental design and timeline for behavioral testing. **G** Representative tracks of mice transfected with Control or Retro-hSyn-Cre+DIO-hM4D(Gi) viruses in the open field test after CPN ligation. **H** Quantification of total distance moved and time spent in the center in the open field test (n = 6 mice for the Control group, n = 7 mice for Retro-hSyn-Cre+DIO-hM4D(Gi) group). **I** Representative tracks of mice transfected with Control or Retro-hSyn-Cre+DIO-hM4D(Gi) viruses in the elevated plus maze test after CPN ligation. **J** Quantification of time in open arms and entries into open arms in the elevated plus maze test (n = 6 mice for the Control group, n = 7 mice for Retro-hSyn-Cre+DIO-hM4D(Gi) group). Data are presented as the mean ± SEM, t-test, $^{*}P$ <0.05, ns, no significance.
